# Cuproptosis: Unraveling the Mechanisms of Copper-Induced Cell Death and Its Implication in Cancer Therapy

**DOI:** 10.3390/cancers16030647

**Published:** 2024-02-02

**Authors:** Chloe Springer, Danish Humayun, Rachid Skouta

**Affiliations:** 1 Department of Biology, University of Massachusetts, Amherst, MA 01003, USA; cospringer@umass.edu; 2 Department of Chemistry, University of Massachusetts, Amherst, MA 01003, USA; dhumayun@umass.edu

**Keywords:** cuproptosis, copper-induced cell death, copper ionophores, oxidative stress, copper homeostasis, lipoylation, elesclomol, mitochondrial respiration, cancer therapy

## Abstract

**Simple Summary:**

Cuproptosis is a recently identified form of cell death induced by imbalanced copper levels. We aimed to explore the mechanism underlying copper-induced cell death. To achieve this, we surveyed the literature to understand the biochemical implications of cuproptosis in diseases, particularly in the context of cancer. The concept of copper ionophores, such as elesclomol and disulfiram, is highlighted as they elevate intracellular copper levels, triggering oxidative stress and ultimately leading to cell death–a potential avenue for cancer therapy. Furthermore, we will delve into the intricate relationship between copper, mitochondrial respiration, and protein lipoylation, shedding light on their connections in inducing cell death.

**Abstract:**

Copper, an essential element for various biological processes, demands precise regulation to avert detrimental health effects and potential cell toxicity. This paper explores the mechanisms of copper-induced cell death, known as cuproptosis, and its potential health and disease implications, including cancer therapy. Copper ionophores, such as elesclomol and disulfiram, increase intracellular copper levels. This elevation triggers oxidative stress and subsequent cell death, offering potential implications in cancer therapy. Additionally, copper ionophores disrupt mitochondrial respiration and protein lipoylation, further contributing to copper toxicity and cell death. Potential targets and biomarkers are identified, as copper can be targeted to those proteins to trigger cuproptosis. The role of copper in different cancers is discussed to understand targeted cancer therapies using copper nanomaterials, copper ionophores, and copper chelators. Furthermore, the role of copper is explored through diseases such as Wilson and Menkes disease to understand the physiological mechanisms of copper. Exploring cuproptosis presents an opportunity to improve treatments for copper-related disorders and various cancers, with the potential to bring significant advancements to modern medicine.

## 1. Introduction

Copper (Cu) is one of the first metals known to be used by humans. Archaeological evidence suggests that its usage dates to 10,000 years ago by ancient civilizations in regions such as Mesopotamia, Egypt, China, and India [1]. Copper was not recognized to have a role in the human body until the 19th century [2]. Researchers began to understand that copper was not just a useful material but an active participant in biochemical processes [2]. 

Today, copper is a well-known inorganic element and ranks as the third most abundant trace element in the body after zinc and iron [3]. Within the average adult body, copper is present within a range of 50–120 mg, with serum concentrations ranging up to 1.5 mg/L [3]. In the United States, the average daily dietary Cu intake is approximately 1 mg [3]. Copper is essential for human health as it is involved in various enzymatic processes and plays crucial roles in many biological processes [4]. Despite supporting vital biologic functions, copper holds a great potential to be toxic and can lead to numerous human diseases once it is dysregulated [5]. Therefore, copper’s homeostatic regulation is under strict control; it is regulated through intestinal absorptive cells as well as excretion through the liver, which then releases extra Cu into bile [6].

Copper can exist in either a reduced (Cu^+^) or oxidized (Cu^2+^) state [5]. Its redox properties make copper both beneficial and toxic to the cell [7]. For instance, the free intracellular Cu during the transition between the Cu^+^ and Cu^2+^ states can produce hydroxyl radicals, namely, reactive oxygen species (ROS) [8]. The creation of ROS can be deleterious to proteins, lipids, DNA, fats, and nucleic acids. Additionally, they can interfere with the production of iron–sulfur clusters, which are essential to many enzymes in our cells [9]. 

**Figure 1:**
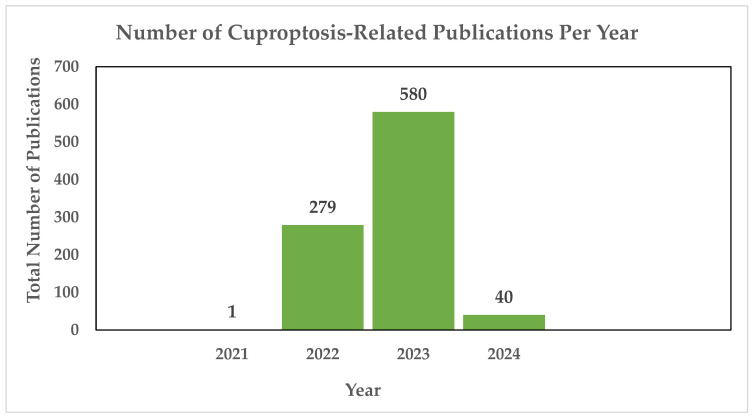
Annual count of cuproptosis-related publications utilizing the PubMED search engine. In 2021, the first cuproptosis-related article was published [11], cited 65 times. In 2022, Tsvetkov and colleagues introduced the concept of a distinct form of regulated cell death induced by copper, known as cuproptosis, cited 844 times. The same year saw a notable surge, with 279 publications exploring cuproptosis, followed by a continued increase in 2023 with 580 publications. As of 2024, an additional 40 publications have been dedicated to the study of cuproptosis thus far [12].

Copper-induced cell death, also known as copper toxicity or cuproptosis, describes the harmful effects that excessive copper levels induce on cellular processes [10]. Cuproptosis is a novel form of cell death, and the scientific community is in the process of unraveling its intricacies. As shown in Figure 1, there have been 900 publications thus far dedicated to the research of cuproptosis, highlighting the ongoing efforts to understand this novelty.

**Figure 2:**
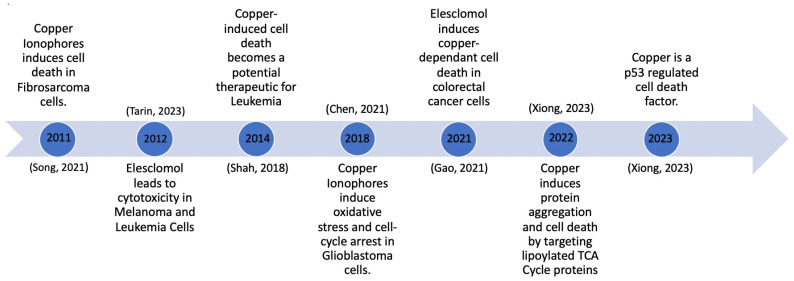
Copper-induced cell death milestones [13,14,15,16,17,18].

One of the more recent advancements in the context of copper-induced cell death is the research and development of copper ionophores. Copper ionophores are molecules to which copper attaches outside of the cell, and they subsequently shuttle it inside the cell. The recent advancements in copper ionophore molecules have made them one of the topics of interest in the development of potential copper therapies. Significant developments occurred between 2011 and 2023, as depicted in Figure 2. This timeline tracks the evolution of copper ionophores, beginning with their testing and induction of cell death in fibrosarcoma cells, to their development as a p53-regulated cell death mechanism (Figure 2). Growing knowledge about copper ionophores suggests that copper-induced cell death could become a therapeutic approach for cancer. An abundance of copper can interfere with cellular membranes, trigger the production of ROS, and disrupt crucial biological functions within cells, ultimately causing cell damage and premature cell death [13].

While cuproptosis shares commonalities in proteins and lipids with other forms of cell death like apoptosis [19], ferroptosis [20], and pyroptosis [21], it also exhibits distinct molecular mechanisms that distinguish it as a distinct form of cell death [22]. Notably, treatments with ferroptosis inhibitor (ferrostatin-1), necroptosis inhibitor (necrostatin-1), and an oxidative stress (N-acetyl cysteine) all were unsuccessful in terminating copper ionophore-induced cell death [22]. Furthermore, cuproptosis stands out as a novel and distinct form of cell death due to its association with mitochondrial respiration and the lipoic acid (LA) pathway [22]. A representation of these distinct characteristics is shown in Table 1.

## 2. Proposed Mechanisms of Cuproptosis

### 2.1. General Mechanisms of Cuproptosis

Many different cellular functions require copper, especially as a cofactor in enzymes to catalyze biology reactions [8]. Therefore, copper is always needed inside a cell at a specific critical concentration [59]. However, if the concentration of copper increases or decreases past the critical concentration, it has been shown to cause cytotoxicity in cells [8]. Additionally, cells have natural homeostatic processes and functions that can bring a cell back to equilibrium if there is an accumulation or decumulation of copper in the cell. Current research efforts are trying to understand how to turn off or break through those processes in hopes of initiating cytotoxicity to cause cell death for cancer treatment. 

To initiate this process, copper must first enter the cell. This can be carried out by copper ionophores which are small molecules that copper binds to be transported into the cell [60]. There are various types of copper ionophores that shuttle copper into the cell. For example, dithiocarbonates, bis(thiosemicarbazone) ligands, 8-hydroxyquinolines, flavones, elesclomol, and disulfiram. Copper ionophores then trigger signaling cascades that are part of the cell death pathway [59]. While the complete mechanism is not well understood yet, it has been pointed out that cells that depend more on mitochondrial respiration, which includes the electron transport chain, are more sensitive to copper ionophores than cells that mostly depend on glycolysis as their main energy [22,59]. It is also understood that copper does not target the electron transport chain, but rather the citric acid cycle (CAC), leading to the conclusion that cuproptosis and mitochondrial respiration are linked [59]. 

Furthermore, genetic screenings were performed to identify the pathways associated with cuproptosis. It was concluded that copper ionophores have two targets, *FDX1,* which codes for a reductase, FDX1, that reduces Cu^2+^ to its more toxic form, Cu^+^, and genes that encode parts of the lipoic acid pathway and protein targets of lipoylation, which are also the key mediators of cuproptosis [22]. Lipoylation is a post translational modification where lipoamide is attached to a lysine residue [59]. If FDX1 and the protein lipoylation process are disturbed, the regulation of copper will be limited; therefore, cytotoxicity of copper can be achieved, leading to cuproptosis. However, the exact mechanism of these genetic encoders and cuproptosis is still being explored [61].

#### 2.1.1. Copper Ionophores

Copper ionophores are shuttlers that help facilitate the movement of copper into cells [9]. Copper ionophores, for example, elesclomol, form a coordination complex with Cu^2+^, which is made up of coordinate covalent bonds between the copper ion and the copper ionophore [62]. In elesclomol, there is a center into which Cu^2+^ fits into and is surrounded by other molecules and ions that help to stabilize the metal ion and its charge, forming a membrane-permeable coordination complex [62]. Once Cu^2+^ enters the mitochondria from the coordination complex, it is reduced to Cu^+^ [11]. While there are many different types of copper ionophores, in this review, we will focus on only two main ionophores that have been studied in relation to cuproptosis, elesclomol and disulfiram [9]. 

#### 2.1.2. Elesclomol

**Figure 3:**
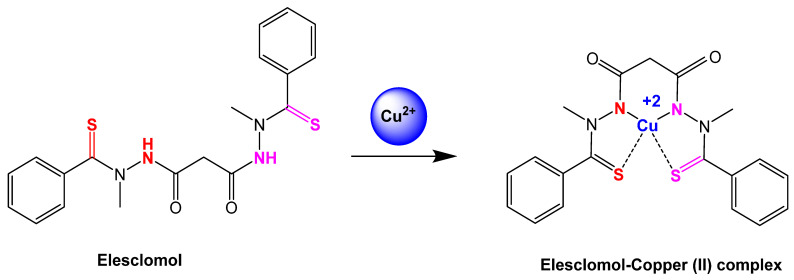
Reaction step of elesclomol chelated with copper (II) ion [64]. Elesclomol is converted to elesclomol-Copper (II) with the addition of copper (II) ion in the extracellular environment.

Elesclomol has been a focus of recent research since it is believed to be at the center of finding out how to induce cuproptosis [60]. Elesclomol is a type of copper ionophore that has a hydrophilic “hole” in the center where Cu^2+^ ions can bind to and enter the cell, as shown in Figure 3 [63]. Elesclomol has also been proven to target cells that are more dependent on oxidative phosphorylation rather than glycolysis, meaning well-oxygenated and mitochondria-dependent cells [59]. This ionophore has also proven to be a strong inhibitor for the growth of cancer cells, thus exhibiting antitumor properties [22].

Introducing elesclomol into a cell triggers cell death through cuproptosis due to excess copper which elevates ROS concentrations, inducing oxidative stress [65]. Oxidative stress arises from the imbalance between the production and accumulation of ROS [66]. Untreated, ROS have detrimental effects on cells, causing damage to DNA, proteins, and lipids [64]. ROS are formed from free radicals of oxygen and are neutralized by antioxidants to prevent cellular damage [66]. 

**Figure 4:**
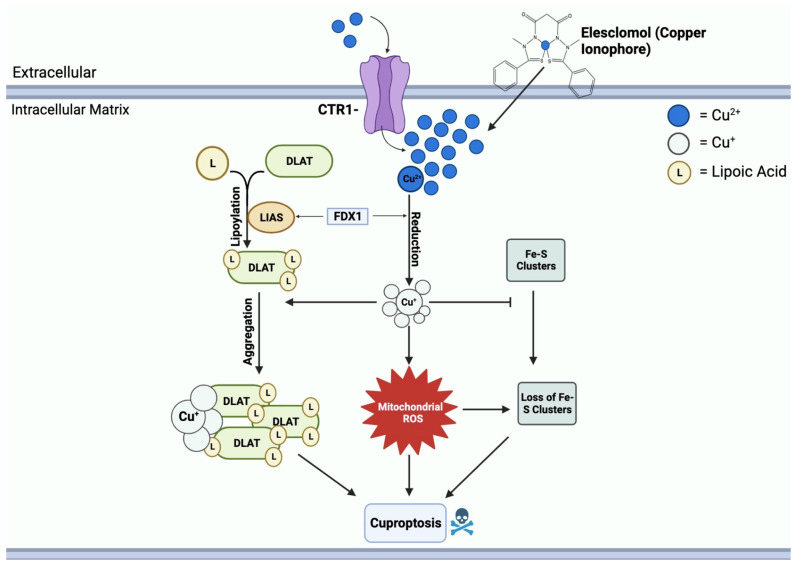
Mechanisms of copper-induced cell death. Elesclomol and CTR1 shuttle Cu^2+^ into the mitochondrial matrix, creating an accumulation of copper. FDX1 reduces Cu^2+^ to Cu^+^, resulting in ROS formation. Cu^+^ and ROS block the synthesis of Fe-S clusters. FDX1 also binds to LIAS, promoting the lipoylation of DLAT. Cu^+^ further binds to lipoylated DLAT and induces aggregation. Mitochondrial ROS, loss of Fe-S clusters, and DLAT aggregation all contribute to cuproptosis cell death.

Elesclomol binds to Cu^2+^ in the extracellular environment and transports it into the mitochondrial matrix [64]. Within the matrix, Cu^2+^ is reduced to Cu^+^ by FDX1, leading to the generation of ROS [64]. FDX1 further interacts with lipoyl synthase (LIAS) to facilitate the lipoylation of DLAT [67]. Moreover, FDX1 regulates the binding of DLAT and copper; Cu^+^ binds to lipoylated mitochondrial proteins, specifically DLAT, triggering proteotoxic stress, aggregation, and eventual cell death [68]. Another crucial function of Cu^+^ is to inhibit the synthesis of Fe-S clusters, essential components of key metabolic enzymes and the electron transport chain [69]. The disruption of Fe-S clusters exacerbates mitochondrial dysfunction linked to cuproptosis, contributing to the cascade of events resulting in cell death [70]. This mechanism of copper-induced cell death through elesclomol is shown in Figure 4. 

#### 2.1.3. Disulfiram

**Figure 5:**
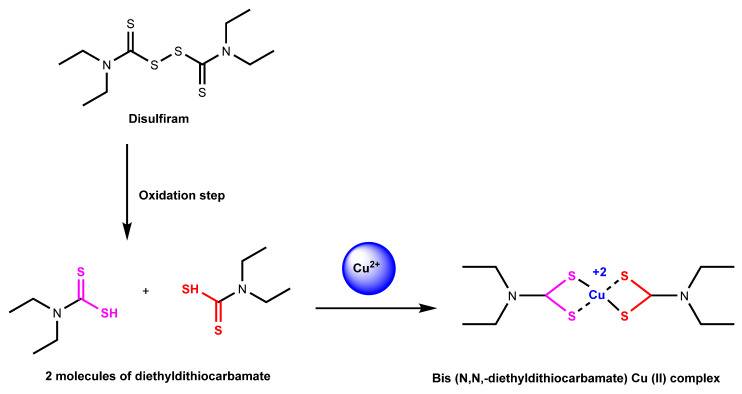
Reaction mechanism of disulfiram chelated with copper (II) ion. Disulfiram is split up into diethyldithiocarbamate, which is then turned into Cu (II) diethyldithiocarbamate with the addition of copper (II) ion in the extracellular environment [73].

Disulfiram is another known copper ionophore that helps shuttle copper into cells. This ionophore has been proven to help in the treatment of alcoholism and suggests that it can be used for antitumor therapies [71]. In addition, several studies have suggested that disulfiram can raise the potency and effects of anticancer drugs [72]. Disulfiram has a known target of proteasomes and helps inhibit the proteasomal mechanism [71]. Proteasomes are a series of enzymes that can break apart certain proteins through the hydrolysis of peptide bonds [71]. The proteasome-mediated degradation pathway is the subject of much anticancer drug therapy research. Disulfiram, when bonded with copper to form a complex, as shown in Figure 5, can become a proteasome inhibitor and targets tumor cellular copper, which can lead to cell death [71].

### 2.2. Ionophores and Chelators Roles in Copper Regulation

**Figure 6:**
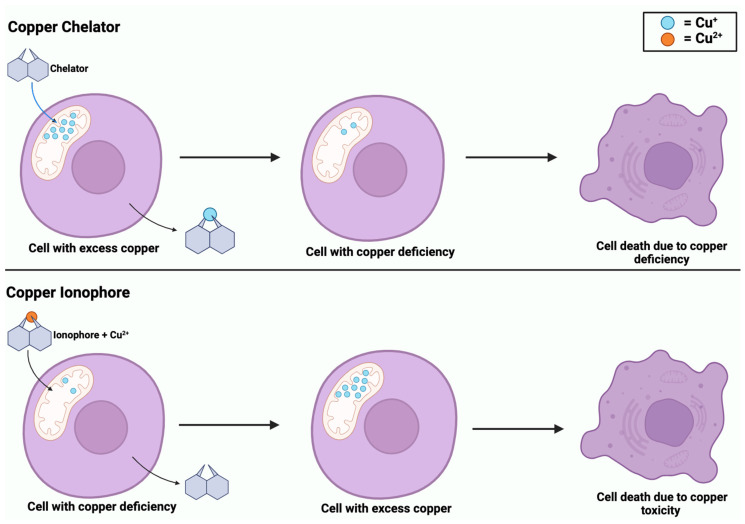
Copper-induced cell death via copper chelators and ionophores. Copper chelators enter the mitochondrial matrix to remove excess Cu^+^ from the cell. Chelators deplete intracellular Cu^+^ levels, inducing a state of copper deficiency. In contrast, copper ionophores enter the mitochondrial matrix with Cu^2+^, causing copper accumulation. Both processes of copper deficiency and accumulation lead to copper-induced cell death.

While copper ionophores and copper chelators share similarities in their involvement in copper regulation, they are distinct compounds. Copper is an element that exists as metallic copper (Cu) and is involved in the homeostasis of biological processes, including cancer [71]. Dysregulation of copper can lead to oxidative stress and cell death through cuproptosis [22]. Copper ionophores are small molecules that form complexes with copper and transport it across the cell membrane, specifically into the mitochondria [22]. The introduction of copper ionophores into a cell induces copper accumulation, which triggers oxidative stress and leads to cuproptosis [71]. Comparatively, copper chelators are compounds that bind to copper ions and remove toxic copper from the cells to prevent its accumulation [5]. These compounds help to balance copper levels within the cell to maintain homeostasis. However, as shown in Figure 6, both compounds can induce copper-dependent cell death, whether due to an excess or deficiency of copper. 

### 2.3. Mitochondrial Respiration and Copper Ionophore Effects

Mitochondria are membrane-bound organelles located in eukaryotic cells and they play a crucial role in the production of metabolic energy [74]. Copper is an essential trace element that serves as a cofactor for numerous enzymes involved in ATP synthesis in the mitochondria [74]. These copper-dependent enzymes play a key role in the final stages of cellular respiration [75]. Cellular respiration generates energy, also known as ATP, through the following steps: glycolysis, occurring in the cytoplasm, the tricarboxylic acid (TCA) cycle, and oxidative phosphorylation [76]. Mitochondrial respiration encompasses the phases of cellular respiration that occur exclusively within the mitochondria: the TCA cycle and oxidative phosphorylation [77]. Oxidative phosphorylation, the final step of cellular respiration occurring within the inner mitochondrial matrix, is responsible for the majority of ATP production in eukaryotic cells by breaking down lipids and carbohydrates to convert them to ATP [78]. Recently, the novelty of copper-dependent cell death being reliant on mitochondrial respiration has been established [22]. Copper ionophores can enhance the activity of lipoylated enzymes within the TCA cycle, pyruvate dehydrogenase (PDH) and alpha-ketoglutarate dehydrogenase (KDH). Lipoylation occurs when a small molecule called a lipoic acid attaches itself to specific enzymes, increasing their activity [79]. When copper ionophores enter the cell, they affect copper homeostasis within the mitochondria and promote the attachment of lipoic acids to these enzymes, making them more active. When cells have elevated lipoylated TCA enzymes, the final products of the TCA cycle are amplified. These products include high-energy electron carriers such as NADH and FADH. These electron carriers are crucial for ATP generation and are the reactants for oxidative phosphorylation [74]. Therefore, when cells have higher levels of lipoylated TCA enzymes, potentially caused by the entry of copper ionophores, there is a greater reliance on mitochondrial respiration for energy production. 

It was also shown that inhibiting the mitochondrial pyruvate carrier, which imports pyruvate from the cytosol to the mitochondrial matrix to use in glycolysis, decreased copper-induced cell death [75]. This supports the crucial role that the PDH complex plays in cuproptosis and how, consequently, an increase in PDH would ultimately enhance mitochondrial respiration alongside copper-induced cell death [75]. Furthermore, the disruption caused by copper ionophores leads to a higher susceptibility of these cells to undergo copper-induced cell death, making these cells that are undergoing mitochondrial respiration 1000-fold more sensitive to copper ionophores [19]. As it is concluded that copper-dependent cell death is dependent on mitochondrial respiration, copper ionophores may hold potential promise as a therapeutic target for conditions where dysregulated mitochondrial function is a contributing factor. 

### 2.4. Lipoylation

Lipoylation is a post translational modification that occurs on four metabolic enzymes: pyruvate dehydrogenase (PDH), acetoin dehydrogenase complex (AoDH), alpha ketoglutarate (KDH), and glycine cleavage system (GCV) [80]. Lipoylation must occur on these proteins that are part of the larger complex for proper enzymatic function [78]. For this mechanism to occur, a lipoamide is covalently attached to a lysine residue [71]. Lysine is added to these proteins to ensure the proper enzymatic functions [22]. Two of the enzymes that require lipoylation are PDH and KDH, both of which regulate the key entry points of carbon into the CAC [71]. PDH is responsible for the conversion of pyruvate into acetyl-CoA during glycolysis before it enters the CAC [71]. Lipoylation is crucial for these enzymes to carry out their normal functions [71].

Gene knockout screening has shown that deletion of LIAS leads to resistance to copper-induced cell death [19]. In addition, it was found that more copper-selective compounds, like elesclomol and disulfiram, were non-functioning when cells were grown in conditions requiring glycolysis [22]. Meanwhile, compounds with the increased ability to attract metal-binding compounds killed cells, without respect to the metabolic state. This result is consistent with a unique characteristic and connection of copper to mitochondrial metabolism-mediated protein lipoylation. Moreover, FDX1 is an upstream regulator of protein lipoylation. This implies that in the absence of FDX1 encoding, protein lipoylation is impeded [78]. It was found that with the FDX1 knockout screening, protein lipoylation did not occur, which led to a drop in cellular respiration as the metabolism of pyruvate to acetyl-CoA could not occur, leading to a halt in cellular respiration [78]. Lastly, there was an increase in the substrate, *S*-adenosylmethionine, of the LIAS in the lipoic acid pathway, which is further evidence of FDX1 being an upstream regulator of protein lipoylation [20]. 

It has been determined that copper toxicity and protein lipoylation are linked [22]; however, no clear direct mechanistic link has been identified yet. Further tests revealed that components of the enzymes requiring lipoylation were found to bind to copper, but not nickel or cobalt [22]. Moreover, it was found that lipoylated TCA cycle proteins binding to copper resulted in a gain of function, leading to cell toxicity [22]. That gain of function resulted in lipoylation-dependent oligomerization of DLAT, a component of one of the targets of lipoylation [81]. Without lipoylation, protein folding into the quaternary structure was dysregulated, further leading to a loss of enzymatic functions in the TCA cycle [79]. Copper is a necessary element for many of the enzymatic processes within the TCA cycle to occur. Without lipoylation with copper, enzymes would not be able to function, leading to a loss of enzymatic function in cell respiration and thus to cell death.

## 3. Copper Relevance in Health and Disease

### 3.1. Copper-Based Nanomaterials

Copper-based nanomaterials (CBNs) have increased in popularity amongst scientists and researchers around the world [82]. Their unique properties and characteristics, particularly their physiochemical features and biocompatibility, make them suitable for biomedical applications such as drug stability, proper biodistribution, improved therapeutic index, and active agent delivery to the specific site [83]. Moreover, CBNs are exceptionally suitable for tumor imaging, as they can make a tumor glow on images of PET, MRI, photoimaging, and CT scans [84]. Tumor imaging and antitumor treatments use CBNs’ strong, almost infrared absorption and photothermal features in photothermal therapy and cancer photoimaging [84]. Beyond their imaging applications, copper-based nanomaterials have become instrumental in antitumor treatments with copper [84]. The large surface area of CBNs proves practical for loading multiple antitumor drugs for targeted cellular entry [84]. Recently, a groundbreaking platelet vesicle-coated cuprous oxide nanoparticle (Cu2O)/TBP-2 cuproptosis sensitization system (PTC) has been developed for effective tumor therapy, particularly in lung metastasis and breast cancer [85]. PTC, designed with enhanced blood circulation and tumor targeting capabilities, releases copper ions and hydrogen peroxides in acidic tumor conditions [85]. Light irradiation induces cuproptosis, inhibiting cancer metastasis and promoting immune response. This study presents a promising nanomedicine design for cuproptosis-based cancer treatment [85]. 

The inherent copper ions in CBNs trigger the production of ROS which increases the cytotoxicity within cells [84]. Upon exposure to light, CBNs trigger a high accumulation of ROS which can be used for photodynamic therapy [86]. In a recent study, a glucose oxidase (GOx)-engineered non-porous copper (I) 1,2,4-triazolate coordination polymer (CP) nanoplatform, denoted as GOx@[Cu(tz)], has been developed for the induction of starvation-augmented cuproptosis and synergistic photodynamic therapy [87]. This design ensures that the catalytic activity of GOx remains shielded within the non-porous scaffold until triggered by glutathione (GSH) stimulation in cancer cells, facilitating targeted cancer starvation therapy [87]. The resultant depletion of glucose and GSH sensitizes cancer cells to GOx@[Cu(tz)]-mediated cuproptosis, leading to the aggregation of lipoylated mitochondrial proteins [87]. In vivo experiments have shown a 92.4% efficacy of tumor inhibition in athymic mice bearing bladder tumors [87]. Notably, this marks the first report of a cupreous nanomaterial capable of inducing cuproptosis and implementing cuproptosis-based synergistic therapy in bladder cancer. 

### 3.2. Anticancer Therapeutic Category

#### 3.2.1. Non-Small Cell Lung Cancer

Lung cancer is the leading cause of cancer death in the U.S. as it accounts for approximately one in five cancer deaths [88]. Specifically, non-small cell lung cancer (NSCLC), which includes squamous cell carcinoma, adenocarcinoma, and large cell carcinoma, is the most common kind of lung cancer, representing 85% of all cases [89]. Notably, smoking, which is a major risk factor, contributes to the formation of malignant lung tissue [90]. In NSCLC, excessive copper accumulation within cancer cells has been closely linked with tumor growth and metastasis [91]. Disrupting copper homeostasis with copper-binding agents like disulfiram has shown promise by enhancing the efficacy of chemotherapy drugs, such as cisplatin, through the induction of oxidative stress and inhibition of DNA mechanisms in lung cancer cells [92]. Recent phase II clinical trials combining disulfiram with cisplatin and vinorelbine demonstrated improved survival rates (10 months vs. 7.1 months for non-disulfiram recipients) in newly diagnosed NSCLC patients, with two long-term survivors identified in the disulfiram group [93]. This finding suggests that integrating disulfiram into NSCLC chemotherapy treatment might enhance patient survival, prompting the need for further exploration in larger-scale trials. Another study investigated the impact of disulfiram and copper on NSCLC growth. The findings suggest that the disulfiram/copper combination induces G2/M phase cell arrest and enhances sensitivity to cisplatin in NSCLC [94]. Initial tests showed that disulfiram or copper separately had a limited impact on NSCLC cell proliferation. Moreover, the combination reduced NSCLC cell colony formation and lung cancer stem cell-related gene expression [95]. 

#### 3.2.2. Colorectal Cancer

Colorectal cancer, also known as colon cancer or rectal cancer, is a malignancy that affects the colon or rectum within the digestive system [96]. Worldwide, it is the third most common cancer, accounting for approximately 10% of all cancer cases, and it is the second leading cause of cancer-related deaths [97]. It is predominantly found in people over the age of 50, and it initiates as small clumps of cells called polyps that form inside the colon [98]. Recently, a novel thieno[3,2-c]pyridine compound called “JFY-001,” designed to target copper ions specifically, demonstrated significant potential in inhibiting colorectal cancer cell proliferation, inducing cuproptosis, and disrupting cellular metabolic processes [99]. When combined with a programmed cell death protein (PD-1) inhibitor, it displayed enhanced antitumor effects, all while exhibiting minimal toxicity to normal cells [99]. Elevated serum copper levels associated with cancer staging and progression in colorectal cancer further underline the significance of targeting copper ions in cancer therapy [100].

#### 3.2.3. Prostate Cancer

Prostate cancer ranks among the most prevalent cancers in men, primarily affecting older individuals, with approximately one in eight men being diagnosed [101]. It originates in the prostate gland, a crucial part of the male reproductive system positioned beneath the bladder and in front of the rectum [102]. Early detection significantly increases the chances of successful treatment as the cancer is confined to the prostate gland. Fortunately, most prostate cancers tend to grow slowly and are low-grade, carrying relatively low risks [103]. However, certain aggressive forms of prostate cancer can rapidly spread throughout the body [103]. In the progression of prostate cancer, there is an observable increase in serum copper levels [104]. A recent study introduces a steroid-based compound that effectively inhibits CTR1 and reduces copper intake in prostate cancer cells, leading to suppressed cell proliferation and progression [105]. This highlights the potential of steroid based CTR1 inhibitors as promising candidates for targeted therapies against cancers dependent on elevated copper levels [105]. Furthermore, copper ionophores have been shown to be an effective therapeutic potential against prostate cancer in vitro and in transgenic adenocarcinoma of mouse prostate (TRAMP) mice, despite unclear molecular mechanisms [106]. Copper ionophore treatments, including disulfiram and clioquinol, elicit toxic levels of ROS specifically within TRAMP cells, while exhibiting no such effect on normal mouse prostate epithelial cells [106].

#### 3.2.4. Uveal Melanoma

Uveal melanoma (UM) is the most common primary intraocular, or eye, malignancy as it occurs in about every five people per million each year globally [107]. There is a lack of effective treatment for UM, and once metastasis occurs, life expectancy can be very poor since most patients of UM succumb to their disease within a year [108]. In studies, it was found that the Cu^+^ accumulation in the UM cells leads to lipoylated proteins aggregating and targeting Fe-S cluster proteins, which leads to cuproptosis [109]. Furthermore, it was found that with the treatment of elesclomol, the viability of the UM cell line M619 decreased, and the ability to migrate and the invasiveness of UM cells were also suppressed [109]. These results yield the conclusion that cuproptosis has a potential therapeutic value for UM patients. Furthermore, recent studies have focused on the relationship between copper-related genes (CRGs) and the prognosis of UM. Three CRGs were tested: ORAI2, ACADSB, and SLC47A, and it was found that all three of these CRGs are unfavorable for the prognosis of UM [110]. Greater expression of these CRGs can lead to an unfavorable prognosis for UM. Furthermore, UM is identified with mutations in GNAQ and GNA11 genes, which are also CRGs, and elesclomol is reported to be a GNAQ/GNA11-specific UM inhibitor [110]. By repurposing elesclomol, it can inhibit the proliferation of cancerous UM cells by targeting GNAQ and GNA11 genes [110]. This conclusion highlights the key connection between copper, CRGs, and the overall outcome of UM.

### 3.3. Copper Chelators in Cancer Therapy

Copper complexes such as copper chelators and copper ionophores have shown promise in cancer targeted therapies [111]. Copper chelators are capable of binding to copper and reducing its bioavailability, thus inhibiting processes such as angiogenesis, which is necessary for tumor growth and metastasis [112]. Certain copper chelation strategies have advanced to clinical trials, including tetrathiomolybdate (TTM) and D-penicillamine (D-Pen) [5]. Moreover, there have been studies where TMM has shown an effect on the reduction in lung, breast, and prostate cancers as well as on squamous cell carcinoma [5]. However, it is important to note that copper chelators are not adequate by themselves to destroy malignant cells and must be combined with other drugs to accomplish a promising therapeutic approach for cancer [113].

### 3.4. Copper Ionophores in Cancer Therapy

**Figure 7:**
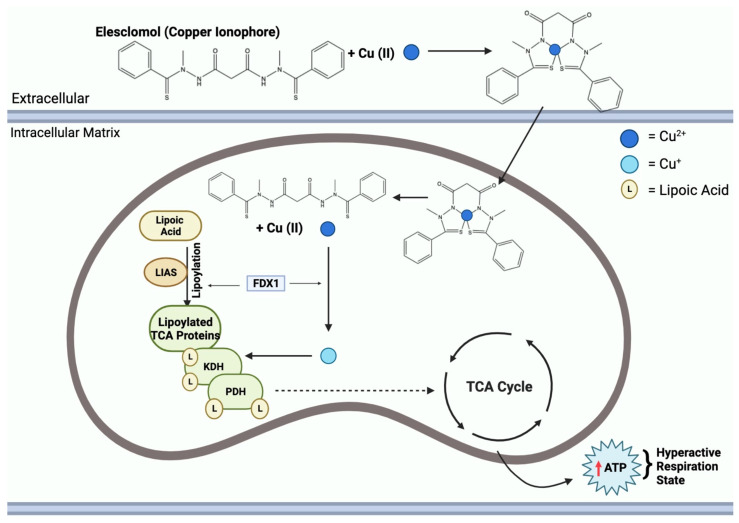
Elesclomol mechanism of action in the mitochondria. Mitochondrial entry of elesclomol carrying Cu^2+^ induces lipoylation of key TCA enzymes, KDH and PDH. Elesclomol-Cu(II) enters the mitochondrial matrix and FDX1 reduces Cu^2+^ to Cu^+^. Additionally, FDX1 facilitates LIAS lipoylation activity of TCA proteins. Cu^+^ binding to lipoylated-TCA enzymes results in a gain of function, leading to the mitochondria being in a hyperactive respiration state, thus resulting in an increase in ATP.

Copper ionophore compounds, including elesclomol, chloroquinol, and disulfiram, are known to increase intracellular copper bioavailability [114]. These compounds release copper within the intracellular environment, triggering various responses. Notably, this leads to an increased production of ROS and restrains proteasome activity in cancer cells, ultimately enabling cell death [5]. Recent research has demonstrated the efficacy of copper ionophores in reducing tumor growth in models of both prostate and breast cancer [110]. As these compounds find applications in therapy, studies have revealed that combining copper ionophores with proteasome inhibitors can stimulate a high mitochondrial state in cells, increasing mitochondrial respiration, posing as an interesting area to study for a possible future application towards tumor therapy [13]. Figure 7 highlights the mechanism of copper ionophores, specifically elesclomol, entering the mitochondria and inducing a hyperactive respiration state. Despite these promising findings, it is important to note that the field of copper metal-binding compounds as cancer therapeutics is still in its early stages of development and requires further exploration.

### 3.5. Elesclomol: An Anticancer Agent

Cancer cells exhibit a remarkable ability to evade the tightly regulated pathways of cellular death, a hallmark feature of their malignancy [115]. This evasion has posed a challenge within cancer therapies as the resistance of cancer cells to programmed cell death pathways diminishes treatment efficacy [116]. However, cuproptosis presents a potential opportunity for overcoming this resistance and promoting cell death in cancer cells. Among the noteworthy developments, the emergence of the Cu^2+^ carrier, elesclomol, has been successful thus far, showing the ability to kill specific drug-resistant cancer cells [54]. This discovery not only emphasizes the potential of cuproptosis as a therapeutic cancer avenue, but it also highlights the tangible progress being made in identifying agents that can effectively target and eliminate cancer cells selectively without harming normal cells [117].

### 3.6. Copper Serum Levels and Cancer

More recently, there has been ongoing research on the connection between copper and cancer [118]. Studies have found that cancer patients have elevated copper levels in both their serum and cancer tissue in a multitude of cancers such as oral, ovarian, gastric, breast, renal, thyroid, esophageal, lung, gallbladder, liver, pancreatic, and prostate cancers [5]. In specific cases, such as colorectal and breast cancer, elevated serum copper levels have been associated with cancer staging and progression [119]. Conversely, decreased serum copper levels have been correlated with colorectal and endometrial cancer [5]. In addition, it has been found that when the tumor was removed, the Cu serum concentrations returned to the normal threshold as seen in healthy patients [5]. Furthermore, copper’s impact on cancer is not limited to its association with serum and tissue levels. It has been found that tumor cells heavily rely on copper for metabolism, and a decrease in or an abundance of copper can have detrimental effects on tumor cells [120].

### 3.7. Copper in Wilson Disease

Wilson disease is a rare genetic disorder that arises from an endogenous accumulation of copper in the body within vital organs, notably the brain, liver, and cornea [121]. Symptoms are predominantly linked to the affected areas, presenting as liver-related issues such as vomiting, yellowish skin discoloration, and leg swelling, or neurological symptoms like tremors, speech difficulties, personality alterations, and auditory or visual hallucinations [121]. This condition follows an autosomal recessive pattern, impacting approximately 1 in every 30,000 individuals [121]. The primary factor contributing to Wilson disease is a mutation in the *ATP7B* gene, a crucial copper transport protein responsible for biliary copper excretion [122]. Mutations in this gene result in the abnormal accumulation of copper in both the liver and brain [30]. Compared to many neurogenetic diseases, Wilson disease responds well to treatment during both its acute and chronic stages [123]. Treatment is lifelong and patients can anticipate a normal life expectancy. The objective of therapy for Wilson disease is to normalize and reduce the concentration of free copper in serum, accomplished through the administration of copper chelators such as D-Pen and trientine [124].

### 3.8. Copper in Menkes Disease

Menkes disease is a lethal X-linked recessive disorder characterized by a deficiency of copper [125]. Initially documented in 1962, this condition now impacts 1 in every 35,000 live male births [126,127]. It originates from a mutation in the *ATP7A* gene, a crucial regulator of copper metabolism in the body [128]. The gene is responsible for facilitating the efflux of copper from absorptive cells, but its dysfunction results in a deficiency of this essential mineral [129]. Menkes disease presents itself with various symptoms, including weakened bones, seizures, kinky hair, and slow physical development [129]. Unfortunately, this rare disease often leads to early childhood mortality. While treatment options exist, such as daily copper injections administrated shortly after birth, there is still difficulty in achieving early diagnosis due to the subtle clinical features and the lack of specific biomarkers [130]. Currently, a definitive cure for Menkes disease remains elusive. However, early intervention with copper–histidine treatment shows promise in ameliorating some of the neurological symptoms associated with the condition [125].

## 4. Targets and Biomarkers

As research on copper ionophores is ongoing, there are yet to be specific biomarkers that have been identified. However, there are proteins that serve as potential biomarkers. These proteins include chaperone proteins (Section 4.1), membrane proteins (Section 4.2), and intracellular proteins (Section 4.3).

### 4.1. Copper Chaperone Protein Biomarkers

Copper chaperones are another type of copper transporters. Their role is to ensure the safe handling and delivery of potentially toxic copper ions to a variety of copper proteins that are essential for copper metabolism. There are various copper chaperones, but some essential ones are ATOX1, CCS, COX17, COX11, and SCO1 (Table 2) [131,132]. First, ATOX1 binds to Cu^+^ and associates with *ATP7A* and *ATP7B*, which makes it part of the copper metalation pathway [131]. Copper chaperone for superoxide dismutase (CCS) is another copper chaperone, and it is responsible for forming heterodimers with copper–zinc superoxide dismutase [133]. Tests have revealed that CCS protein concentrations are higher in many cells that are found in copper-deficient mammals [132]. Thus, it can be a potential biomarker for copper level status. COX17 is another chaperone for copper and it is widely believed that most mitochondrial copper is delivered by this protein [132]. COX17 is 1 of 30 assembly factors necessary for the formation of active cytochrome *c* oxidase (CCO) and is believed to transfer copper from the cytoplasm into mitochondria [92]. COX11, like COX17, is another copper chaperone which is also 1 of 30 assembly factors for CCO91. COX1, a subunit of CCO, receives most of its copper from COX11. SCO1 and SCO2 are two more copper chaperones that deliver copper directly to the copper-binding site of COX2 and control the homeostasis of copper within cells [132]. When copper enters the intestines, specifically the duodenum, the first transporter it interacts with is CTR1 (Table 2) [132]. It is widely believed that CTR1 is the main protein responsible for the transport of copper across the microvilli layer in the duodenum [132]. Through the CTR1 protein, copper can enter the bloodstream and cells. CTR2 is another protein that plays a role in copper import and intracellular copper homeostasis. However, the regulation of copper by CTR2 has not been published yet [132].

### 4.2. Membrane Protein Biomarkers

Divalent metal transport 1 (DMT1) (Table 2), also known as *SLCA112*, is a membrane protein that is associated with iron transport; however, it can transport both forms of copper, Cu^2+^ and Cu^+^ [134]. This import of copper occurs under certain conditions, such as a deficiency in CTR1 (Table 2) [115]. Another membrane-bound protein, amyloid precursor protein (APP) (Table 2), primarily known for its involvement in neurobiology and neurodegenerative disorders could serve as a potential biomarker for copper ionophores and compels further investigation [135]. Given copper’s relevance to neuronal function as well as its association with neurodegenerative diseases, it is important to assess if copper ionophores impact APP expression, processing, or metabolism. With its relation to copper, APP is involved in copper homeostasis, and studies have found that an over expression of APP decreases copper levels in the brain [131]. Experimental studies using cellular models could shed light on more potential connections between copper ionophores and APP.

### 4.3. Intracellular Protein Biomarkers

Intracellular proteins are being explored as potential biomarkers for copper ionophores, revealing insights into the intricate network of copper metabolism within cells. Metallothionein (MT) (Table 2), known for its role in storage and chaperoning, serves as a protein of interest [136]. MT responds to high copper exposure by enhancing copper entrapment and subsequent loss upon cell shedding in the intestine. Copper triggers MT transcription, but its viability as a biomarker requires further study [137]. The copper metabolism MURR domain, represented by COMMD1, is crucial for hepatic copper efflux; it is also tightly linked to *ATP7B* interaction, a copper-transporting protein [138]. COMMD1 also interacts with the X-linked inhibitor of apoptosis (XIAP), suggesting a greater influence [139]. XIAP is essential for COMMD1 degradation as it contributes to normal hepatic copper efflux through its interaction with *ATP7B* [140]. Excess copper in the liver accelerates XIAP catabolism [131]. Furthermore, COMMD1 (Table 2) expression with changes in copper levels have yet to be studied; further research could offer insights into its potential as a biomarker in copper ionophores [131].

**Table 2:** Targets and biomarkers of copper ionophores related to cuproptosis.

Name	Type of Protein	Function	Reference
Antioxidant 1 Copper Chaperone (ATOX1)	Chaperone	Binds to Cu^+^; part of copper metalation pathway	[131,132,141]
Copper Chaperone for Superoxide Dismutase (CCS)	Chaperone	Forms heterodimers with copper–zinc superoxide dismutase	[131,132,142]
Cytochrome C Oxidase Copper Chaperone (COX17)	Chaperone	Delivers mitochondrial copper	[131,132]
Cytochrome C Oxidase Copper Chaperone (COX11)	Chaperone	Delivers copper to COX1	[132]
Synthesis of Cytochrome C Oxidase 1 (SCO1 and SCO2)	Chaperone	Delivers copper to COX2 and helps maintain copper homeostasis	[131,132,143]
High-Affinity Copper Uptake Protein 1 (CTR1)	Chaperone	Transports copper across the lining of the duodenum	[131,132,144]
High-Affinity Copper Uptake Protein 2 (CTR2)	Chaperone	Helps in copper import and copper homeostasis	[131,132,145]
Divalent Metal Transporter 1 (DMT1)	Membrane	Transports Cu^2+^ and Cu^+^	[132]
Amyloid Precursor Protein (APP)	Membrane	Involved in copper homeostasis in the nervous system	[132]
Metallothionein (MT)	Intracellular	Controls high copper levels through copper entrapment and cell shedding	[132,146]
Copper Metabolism Domain-Containing 1 (COMMD1)	Intracellular	Involved in hepatic copper homeostasis	[132]

## 5. Conclusions and Perspectives

The study of cuproptosis and its potential implications in cancer therapy paves a promising avenue for further research and clinical applications. As research progresses, several key directions can be pursued. Molecules, proteins, and signaling cascades involved in cuproptosis and cellular defense responses against copper-induced damage, as well as genetic and epigenetic factors that could influence one’s susceptibility to this novel form of cell death, could be explored. Additionally, to facilitate the advancement of clinical trials and research, the identification of sensitive copper-dependent biomarkers as well as copper ionophore biomarkers becomes crucial [147]. Exploring the changes in mRNA expression and or the protein abundance of copper transporters in response to dietary copper intake could provide valuable insight for potential biomarkers [5]. Moreover, enhancing the selectivity of copper ionophores for cancer cells, as they are currently in the developmental stage for cancer-targeted therapy, holds great potential for improving their clinical efficacy and reducing off-target effects [112]. However, before any meaningful progression can occur in linking cuproptosis and copper to targeted cancer therapy, the regulatory mechanisms underlying cuproptosis must be determined [5]. Finally, the correlation between cuproptosis and cancer needs further exploration, emphasizing the significance of clinical trials that focus on targeting copper in cancer therapy [9]. As the research community aims to understand the intricacies of cuproptosis, its progression holds potential for novel therapeutic strategies that could improve cancer treatment.

## Figures and Tables

**Table 1:** Comparison of cell death pathways.

Cell Death Pathway	Induction Mechanisms	Main Proteins/Genes Involved	Disease Implications
Cuproptosis (discovered in 2022)	Disruption of copper homeostasis, and oxidative stress	FDX1 [23] DLAT [23] DBT [23] GCSH [23] DLST [23] LIAS [23] CTR1 [23] *ATP7A*/*ATP7B* [24] MT [24] GSH [24] PDH [25] PDHA1 [25] PDHB [25] KDH [26] SLC31A1 [27,28] LIPT1 [29] DLD [30]	Neurological diseases (e.g., Menkes and Wilson) [26]. Cancer (e.g., elevated serum copper levels in oral, gallbladder, liver, breast, esophageal, pancreatic, bladder, renal, prostatic, thyroid, cervical, and lung; decreased serum copper levels in endometrial and colorectal) [10,31].
Ferroptosis (discovered in 2012)	Iron-dependent lipid peroxidation	GPX4 [32,33] GSH [34] DMT1 [16] Ferritin [35,36] 15-Lox [17]	Neurodegenerative diseases (e.g., Alzheimer’s and Parkinson’s) [37]. Cancer (e.g., lung, colorectal, prostate, breast, melanoma, hepatocellular carcinoma, pancreatic, and skin) [18,38,39].
Apoptosis (discovered in 1972)	Caspase cascades trigger and regulate programmed cell death	Caspase-3 [40] Caspase-6-10 [41] BCL-2 [42,43] Cytochrome C [44] Apaf-1 [45] CD95, CD95L [46] CAD [47]	Cancer (e.g., systemic breast, lung, kidney, ovary, uterus, melanoma, leukemia, and lymphomas) [48]. Autoimmune disorders (e.g., systemic lupus erythematosus, rheumatoid arthritis, and thyroiditis) [49]. Neurodegenerative diseases (e.g., Alzheimer’s, Parkinson’s, and Huntington’s) [41].
Pyroptosis (discovered in 1986)	Inflammasome activation triggers caspase cascade, leading to programmed cell death	Caspase-1 [50] Caspase-11 [49] IL-1β [49] IL-18 [49] GSDMD [51,52] NLRP3 [53]	Inflammatory disease (e.g., sepsis and Crohn’s) [54,55]. Autoimmune disorders (e.g., arthritis, psoriasis, and dermatitis) [56]. Microbial infection (e.g., legionella, salmonella, and francisella) [57]. Cancer (e.g., colorectal, gastric, and hepatocellular carcinoma) [58].

## Abbreviations

ATP7A	ATPase copper transporting alpha
ATP7B	ATPase copper transporting beta
CTR1	High affinity copper uptake protein 1
CTR2	High affinity copper uptake protein 2
FDX1	Ferredoxin 1
DLAT	Dihydrolipoamide-S-acetyltransferase
MT	Metallothionein
PDH	Pyruvate dehydrogenase
KDH	Ketoglutarate dehydrogenase
CAC	Citric acid cycle
GPX4	Glutathione peroxidase 4
GSH	Glutathione
DMT1	Divalent metal transporter 1
15-Lox	Arachidonate-15-lipoxygenase-1
BCL-2	B-cell lymphoma 2
Apaf-1	Apoptotic protease activating factor 1
CD95	Cluster of differentiation 95 (Fas or APO-1)
CD95L	Cluster of differentiation 95 ligand (FasL)
LIAS	Lipoic acid synthetase
CAD	Carbamoyl-phosphate synthetase 2
GSDMD	Gasdermin D
NLRP3	NLR family pyrin domain containing 3
IL-1β	Cytokine interleukin-1β
IL-18	Cytokine interleukin-18
SLC31A1	Solute carrier family 31 member 1
LIPT1	Lipoyltransferase 1
DLD	Dihydrolipoamide dehydrogenase
DBT	Dihydrolipoamide branched chain transacylase E2
GCSH	Glycine cleavage system protein H
DLST	Dihydrolipoamide S-succinyltransferase
PDHA1	Pyruvate dehydrogenase E1 subunit alpha 1
PDHB	Pyruvate dehydrogenase E1 subunit beta
NADH	Nicotinamide adenine dinucleotide (reduced form)
FADH	Flavin adenine dinucleotide (reduced form)
TTM	Tetrathiomolybdate
D-Pen	D-penicillamine
ATOX1	Antioxidant 1 copper chaperone
CCS	Copper chaperone for superoxide dismutase
CCO	Cytochrome c oxidase
COX17	Cytochrome c oxidase copper chaperone
COX11	Cytochrome c oxidase copper chaperone
SCO1	Synthesis of cytochrome c oxidase 1
SCO2	Synthesis of cytochrome c oxidase 2
APP	Amyloid precursor protein
COMMD1	Copper metabolism domain containing 1
XIAP	X-linked inhibitor of apoptosis
SLCA112	Solute carrier family 11 member 2
UM	Uveal melanoma
